# HOW WIDOWHOOD SHAPES THE EFFECT OF ADULT CHILDREN’S EDUCATION ON MOTHERS’ PSYCHOLOGICAL WELL-BEING

**DOI:** 10.1093/geroni/igad104.3034

**Published:** 2023-12-21

**Authors:** Robert Frase, Shawn Bauldry, J Jill Suitor, Megan Gilligan

**Affiliations:** Southern Illinois University, Carbondale, Illinois, United States; Purdue University, West Lafayette, Indiana, United States; Purdue University, West Lafayette, Indiana, United States; University of Missouri, Columbia, Missouri, United States

## Abstract

Despite a growing interest in the effects of adult children’s education on older parents’ health and well-being, few studies have focused on how family structure may shape these processes. Drawing on data collected from 160 married and 180 widowed mothers ages 73-85 as part of the Within-Family Differences Study, we explore how mothers’ widowhood status moderates the association between their adult children’s education and mothers’ depressive symptoms. Drawing on past empirical work and Socioemotional Selectivity Theory, we develop and test two competing hypotheses. First, empirical research on depression and financial strain suggests that widowed mothers may be especially reliant on their adult children’s resources and, therefore, that adult children’s education would have a larger impact on widowed than married mothers’ mental health. Alternatively, principles of Socioemotional Selectivity Theory suggest that widows may place less value on their children’s material accomplishments, like educational attainment, and instead emphasize the socioemotional aspects of their relationships with their children. Therefore, children’s education may exert a smaller influence on the psychological well-being of widowed than married mothers. Results of linear regression models stratified by widowhood status supported our second hypothesis. Specifically, the association between adult children’s education and mothers’ psychological well-being was significantly larger among married mothers relative to widowed mothers. Our findings highlight the importance of considering family processes and family structure when studying educational attainment and health across generations.

